# Editorial: Patient-centered care: strengthening trust and communication in healthcare relationships

**DOI:** 10.3389/fmed.2026.1858637

**Published:** 2026-05-08

**Authors:** Kristen Miller, Yan Xiao, Olga Catherina Damman, Enid Montague, Kelly Smith

**Affiliations:** 1 MedStar Health, Columbia, MD, United States; 2 The University of Texas at Arlington, Arlington, VA, United States; 3 Universiteit van Amsterdam, Amsterdam, Netherlands; 4 University of Toronto, Toronto, ON, Canada

**Keywords:** communication, patient-centered care, socioecological model, systems engineering, trust

## Introduction

Patient-centered care is often described as a guiding value in healthcare, yet in practice it is most clearly experienced through trust and communication (1). A growing body of work highlights that patient–physician communication and active patient involvement are central to care, shaping both patient experience and downstream health outcomes, particularly in how care decisions are made and understood Hendrix et al. (2–8). The patient-physician relationship is at the core of patient-centered care with direct and indirect pathways to impact health outcomes (9, 10), including medication errors (11). Specific techniques have been proposed to improve patient-physician communication, such as agenda setting, Shared Decision Making and minimizing interruptions (12, 13). Key considerations in patient-physician communication include respect, empathy, transparency, and direct face-to-face interactions (14). Trust shapes whether patients feel heard, whether they share critical information, and whether they follow through on care, while communication is the primary mechanism through which that trust is built or eroded. Importantly, neither trust nor communication exists in isolation. They are influenced not only by what happens in a single clinical encounter, but also by the broader context within which care is delivered.

The socioecological model (SEM) provides a useful framework for understanding this complexity, emphasizing that health experiences are shaped across multiple, interconnected levels, including individual, interpersonal, community, and societal factors (15–18). At the individual level, this includes patients' knowledge, beliefs, health literacy and prior experience. Especially the ability to access, understand, appraise, and use information and services is also reflected in the concept of health literacy, or “the personal knowledge and competencies of individuals that accumulate through daily activities, social interactions and across generation.” At the interpersonal level, relationships with clinicians and caregivers; at the community level, the social and cultural contexts in which care occurs; and at the societal level, broader structural forces such as policy and access to resources. Applied to patient-centered care, this perspective highlights how trust and communication operate across these interacting levels, from the micro-level of patient–clinician interactions, to meso-level care teams and organizations, to macro-level systems, structures, and social conditions.

The Research Topic, patient-centered care: strengthening trust and communication in healthcare relationships, brings together 23 articles spanning diverse settings and populations, illustrating how trust and communication within patient-centered care are shaped across these levels, from individual experiences and interpersonal communication to organizational structures and broader societal conditions, and how improving health requires alignment across them.

## Individual level—Patient voice, beliefs, preferences, and lived experience

At the individual level, patient-centered care is shaped by patients' knowledge, beliefs, preferences, prior experiences, and capacity to engage in their care (19). These studies highlight how these factors influence patients' information needs, decision-making preferences, and how patients interpret, respond to, and participate in healthcare. This work shows that trust and communication begin before any clinical interaction takes place, underscoring the need for communication approaches that are responsive to individual context, capacity.

Hendrix et al. leveraged a co-design approach to develop patient education interventions aimed at improving medication safety, identifying knowledge gaps and information asymmetry as key barriers. They found that patients showed greater interest in partnership-focused educational content and that addressing information asymmetry may support more active patient engagement and shared decision-making. Fusiak et al. found that preferences for involvement in care are not fixed and may vary depending on how they are elicited, pointing to the importance of using thoughtful methods to understand what role patients want to play in decision-making. Extending this focus beyond patients alone, the review by Ofosu et al. captured the emotional, ethical, and practical complexity caregivers face when making high-stakes decisions under uncertainty, showing how limited support and power imbalances can shape participation and trust. Finally, Zhang, Teng et al. provided a window into the lived realities of managing chronic illness, illustrating how ongoing physical, emotional, and social burdens shape how individuals experience care and interact with healthcare providers.

## Interpersonal level—Partnership, participation, and the clinical encounter

At the interpersonal level, patient-centered care is enacted through relationships between patients, clinicians, and caregivers, where communication serves as the primary vehicle for care delivery. These studies focus on the dynamics of these interactions, including how communication strategies and behaviors of both patients and clinicians, as well as relational cues, influence understanding and engagement. Together, they demonstrate that trust is built through interaction, not intention. Both verbal and non-verbal communication, as well as respect, responsiveness, and inclusion in decision-making (i.e., Shared Decision Making), shape whether patients feel heard, valued, and confident in their care, ultimately influencing satisfaction, adherence, and outcomes.

In an opinion piece, Faytong-Haro examined how patients' symptoms and concerns may be dismissed or minimized across clinical contexts such as chronic, poorly understood conditions, mental health, and women's health, contributing to emotional distress, delayed diagnosis, and erosion of trust, particularly among marginalized populations. The author concludes that these patterns are driven by a combination of information asymmetry, bias, and systemic constraints, and emphasizes the need for improved communication, clinician awareness, and system-level changes to rebuild trust and support safer care. The review by Piatkowska et al. highlighted both the promise and challenges of implementing Shared Decision Making in the context of asthma and chronic respiratory disease management, emphasizing the need for approaches that are feasible and responsive to real-world clinical settings. Efforts to better capture and evaluate communication are reflected in the original research by Shao et al., which introduced a structured, patient-reported scale to assess doctor–patient communication quality, including how communication is experienced and its impact on engagement and health behaviors.

A collection of original research studies further illustrates how communication strategies within the clinical encounter must adapt to patient context and preferences. Zheng and Lin found patient expectations and capacity to engage vary widely, requiring adaptable communication approaches, while Chen et al. reinforced the role of trust and respect as foundational to effective decision-making. From the patient perspective, Jameel et al. links communication quality to overall satisfaction and trust, and Cano et al. highlights how competing priorities and structural challenges shape communication in complex care contexts. Finally, Schneider et al. demonstrates how even subtle non-verbal cues can influence patient perceptions and satisfaction, reinforcing that communication extends beyond words alone.

## Community and organizational level—Context, culture, and conditions of care

At the community and organizational level, patient-centered care is shaped by the environments in which care is delivered, including care delivery models, access to services, and the broader social and cultural context. These studies highlight how care delivery structures, continuity, and community-based resources shape the context in which patient–clinician communication occurs. Collectively, this work illustrates that trust and communication are not solely the responsibility of individual clinicians but are enabled or constrained by system design. Access, coordination, and organizational culture shape the conditions under which meaningful communication can occur and ultimately determine whether patient-centered care can be consistently delivered.

At the level of care delivery experience shaped by organizational context, Zhang, Wang et al. demonstrated that how patients experience and evaluate care strongly influences whether they engage with and consistently use primary care services. Similarly, in a brief research report, Moreno and Cornejo highlighted the importance of sustained relationships in care among older adults in primary care settings, linking continuity not only to patient experience but also to broader wellbeing, including mental health outcomes. Expanding beyond individual encounters, Mitnik et al. illustrated how community-based oncology centers can improve access to care by bringing services closer to patients' homes, while also highlighting the need for strong coordination between community and hospital teams to maintain patient confidence, ensure safety, and support continuity of care.

At the organizational level, Alshahrani et al. demonstrated that workforce experience directly influences communication quality and patient trust, with supportive work environments enabling more effective and patient-centered interactions. Complementing this systematic review, the brief research report by Reid et al. highlighted how workflow integration, competing demands, and technological fit shape whether new tools can be successfully implemented. Together, these studies reinforce that patient-centered care depends not only on what clinicians do, but on how systems are designed to support consistent, coordinated, and meaningful communication and relationships over time.

## Policy and societal level—Systems, structures, and the design of care

At the policy and societal level, patient-centered care is influenced by broader structural forces, including socioeconomic conditions, cultural norms, and health system policies that shape access to care and distribution of resources. These studies highlight how disparities and structural barriers impact patient experiences across settings and populations. Taken together, they emphasize that trust in healthcare providers, systems, and the care patients receive is deeply tied to equity and fairness. When patients encounter systemic barriers, discrimination, or inconsistent access, trust can be eroded before any interaction occurs, reinforcing the need to address structural determinants as part of delivering truly patient-centered care.

At a global level, Bhatt et al. highlight how cultural and socioeconomic factors shape family satisfaction with care in intensive care unit (ICU) settings through a systematic review, finding that communication quality, support for shared decision-making, and attention to family needs are key drivers of satisfaction, while gaps remain in understanding these dynamics in low- and middle-income countries. Similarly, Kong et al. illustrate how geographic isolation, social context, and resource constraints shape interpersonal health communication among older adults in rural China, demonstrating that communication is often centered on face-to-face interactions with family doctors and influenced by multilevel factors, including health literacy, provider workload, and broader policy and environmental conditions.

At the intersection of structural context and patient experience, García-Sáenz et al. explored how patients with HER2+ metastatic breast cancer navigate uncertainty and transitions across care contexts, revealing how health system structures and information delivery practices, rather than individual factors alone, shape communication, expectations, and engagement. As an example of how system-level design can address structural barriers, Cohen et al. demonstrates how proactive, relationship-based outreach models like their novel Boost Team can be embedded within care systems to improve access, connect patients to needed services, and build trust among underserved populations. Together, these studies highlight that improving patient-centered care requires not only better communication in the clinical encounter, but intentional redesign of the structural conditions that shape when, how, and whether communication and trust can occur at all, including efforts to improve access and reduce disparities.

## Innovation, technology, and new frontiers in communication

Spanning multiple levels of the SEM, emerging technologies are increasingly shaping how patients and clinicians communicate and engage in care. These studies explore how tools such as imaging and artificial intelligence can influence understanding, transparency, and interaction. Collectively, this work highlights that technology has the potential to both strengthen and disrupt trust in healthcare providers, systems, and the tools themselves. When thoughtfully implemented, it can enhance communication, identify gaps, and empower patients, but without careful integration, it risks introducing new barriers or reinforcing existing inequities.

Through their mini review, Conte et al. demonstrate how visual tools and imaging modalities in inflammatory arthritis care can enhance patient understanding, engagement, and treatment adherence by making disease processes more visible and accessible. Complementing this, Zhou et al. analyzed clinical transcripts from primary care encounters to identify communication gaps, finding that while diagnostic accuracy may remain high, important contextual and patient-specific details are frequently missed, and that empathy and clear explanations are strongly associated with higher patient satisfaction. Together, these studies suggest that technology can not only improve information exchange but also serve as a mechanism for identifying gaps and supporting reflection, advancing more complete, transparent, and patient-centered communication while requiring thoughtful integration to maintain trust in clinicians, systems, and the technologies themselves.

## Conclusion

Together, the studies in this Research Topic demonstrate that patient-centered care is not a single intervention or approach, but a system-level outcome that emerges from alignment across the SEM. Trust and communication are shaped at every level, from patients' knowledge, preferences, and lived experiences, to interpersonal interactions with clinicians, to the design of care environments, and to broader structural conditions that influence access and equity. When these levels are aligned, clinician-patient communication is more effective, relationships are stronger, and patients are better able to engage in their care. When they are not, even well-intentioned efforts can fall short, resulting in fragmented communication and weakened trust across the care experience.

This body of work reinforces that improving patient-centered care requires moving beyond a narrow focus on individual clinician behaviors to a broader, systems-oriented perspective (20). Prior studies consistently show that communication and trust are shaped not only within clinical encounters, but also by how care is organized, how information is delivered, and how patients navigate healthcare systems over time. However, gaps remain in translating these insights into practice. Existing literature identifies multilevel influences on communication and trust, but provides limited guidance on how to design, implement, and sustain interventions that operate across these levels and are responsive to diverse patient contexts. There is also a need to better understand how to repair trust once it has been eroded, how to ensure communication remains effective in increasingly complex care environments, and how emerging technologies influence both trust and understanding over time. While many traditional strategies have focused on clinicians, newer approaches—such as digital and patient-facing tools—offer opportunities to better support patients and families as active partners in care (21). Ultimately, advancing patient-centered care will depend on intentionally designing for trust and communication as core components of care delivery across the full healthcare experience.
